# Activation of Shc1 Allows Oncostatin M to Induce RANKL and Osteoclast Formation More Effectively Than Leukemia Inhibitory Factor

**DOI:** 10.3389/fimmu.2019.01164

**Published:** 2019-05-28

**Authors:** Emma Persson, Pedro P. C. Souza, Thais Floriano-Marcelino, Howard Herschel Conaway, Petra Henning, Ulf H. Lerner

**Affiliations:** ^1^Department of Molecular Periodontology, Umeå University, Umeå, Sweden; ^2^Bone Biology Research Group, Department of Physiology and Pathology, School of Dentistry, São Paulo State University (UNESP), Araraquara, Brazil; ^3^School of Dentistry, Federal University of Goiás, Goiânia, Brazil; ^4^Department of Physiology and Biophysics, University of Arkansas for Medical Sciences, Little Rock, AR, United States; ^5^Department of Internal Medicine and Clinical Nutrition, Centre for Bone and Arthritis Research, Institute for Medicine, Sahlgrenska Academy, University of Gothenburg, Gothenburg, Sweden

**Keywords:** OSM, LIF, RANKL, Shc1, osteoclast, bone resorption

## Abstract

**Background and Purpose:** The gp130 family of cytokines signals through receptors dimerizing with the gp130 subunit. Downstream signaling typically activates STAT3 but also SHP2/Ras/MAPK pathways. Oncostatin M (OSM) is a unique cytokine in this family since the receptor (OSMR) activates a non-redundant signaling pathway by recruitment of the adapter Shc1. We have studied the functional relevance of Shc1 for OSM-induced bone resorption.

**Experimental Approach:** Osteoblasts were stimulated with OSM and STAT3 and Shc1 activations were studied using real-time PCR and Western blots. The role of STAT3 and Shc1 for OSM-induced RANKL expression and osteoclast formation was studied by silencing their mRNA expressions. Effects of OSM were compared to those of the closely related cytokine leukemia inhibitory factor (LIF).

**Key Results:** OSM, but not LIF, induced the mRNA and protein expression of Shc1 and activated phosphorylation of Shc1 in the osteoblasts. Silencing of Shc1 decreased OSM-induced activation of STAT3 and RANKL expression. Silencing of STAT3 had no effect on activation of Shc1, but prevented the OSM-mediated increase of RANKL expression. Silencing of either Shc1 or STAT3 in osteoblasts decreased formation of osteoclasts in OSM-stimulated co-cultures of osteoblasts and macrophages. In agreement with these observations, OSM was a more potent and robust stimulator than LIF of RANKL formation and bone resorption in mouse calvariae and osteoclast formation in bone marrow cultures.

**Conclusions and Implications:** Activation of the Shc1-dependent STAT3 signaling is crucial for OSM-induced osteoclast formation. Inhibition of Shc1 is a potential mechanism to specifically inhibit OSM-induced bone resorption.

## Introduction

Oncostatin M (OSM) belongs to the gp130 family of cytokines. It was discovered as a cytokine released from macrophage differentiated U-937 histiocytic lymphoma cells that inhibited proliferation of melanoma cells (1). OSM has also been reported to be expressed in monocytes, dendritic cells, T-cells, neutrophils (2), intestinal stromal cells (3), osteoblasts (4, 5) and osteocytes (5). Several studies have shown that OSM is involved in a wide variety of functions (2, 6), including bone remodeling (7), embryologic development, liver regeneration, haematopoiesis (8), tumorigenic progression and metastasis formation (9, 10), as well as inflammatory processes such as pulmonary fibrosis (11, 12), asthma (13), inflammatory bowel disease (3), periodontal disease (14), rheumatoid arthritis (15) and neurogenic heterotopic ossifications (16).

Cytokines in the gp130 family bind to cell surface receptor (R) subunits, and the ligand-receptor complex interacts with the transmembrane protein gp130 for signal propagation. Activation of the OSMR triggers heterodimerization between the ligand-receptor complex and one gp130 subunit (2). Human OSM can induce signaling through both the OSMR and the receptor for leukemia inhibitory factor (LIF), a closely related cytokine in the gp130 family, whereas mouse OSM acts mainly through the OSMR:gp130 heterodimer (2, 6), although it has been shown that mouse OSM can stimulate bone formation by decreasing sclerostin expression after LIFR-induced activation of STAT3 (5).

OSM stimulates bone resorption in organ cultures (17) and enhances osteoclast formation in crude bone marrow cell cultures (18, 19), effects which are associated with increased expression of receptor activator of NF-κB ligand (RANKL) (17, 18). Interestingly, OSM is more potent and effective than LIF as a stimulator of osteoclast formation in co-cultures of mouse osteoblasts and bone marrow cells (20). The bone phenotype of mice in which the *Osm* gene has been deleted has not been reported, but mice globally deficient in the *Osmr* have increased bone mass and a decreased number of osteoclasts (5), findings which are in agreement with *in vitro* observations showing OSM increasing osteoclast numbers and stimulating bone resorption.

The OSMR has no intrinsic tyrosine kinase activity, but dimerization with gp130 activates the JAK-STAT (Janus kinase and signal transducer and activator of transcription) pathway. JAKs are constitutively connected to the membrane-proximal regions of gp130 and OSMR and, upon activation, JAKs trans-phosphorylate several Tyr residues in the intracellular domains of gp130 and OSMR. In the mouse OSMR, JAK2 is preferentially bound and its activation leads to phosphorylation of Tyr^917^ and Tyr^945^ in the OSMR and subsequent recruitment of the transcription factor STAT3 (21, 22). Recruitment of STAT3 to gp130 is mediated by JAK-dependent phosphorylation of Tyr^767^/Tyr^814^/Tyr^905^/Tyr^915^ (23). Once phosphorylated by JAKs, activated STAT3 dimers translocate to the nucleus and bind to specific DNA sequences in promoter regions of a variety of target genes. JAK-dependent phosphorylation of Tyr^759^ in gp130 results in recruitment and activation of the tyrosine phosphatase SHP-2 [Src homology region 2-containing protein tyrosine phosphatase 2; (24)]. In turn, SHP-2 then forms a complex with Grb2 (growth factor receptor-binding protein 2) and Sos (Son of sevenless), which activates the Ras/Raf/MAPK pathway (25), a hallmark of many haematopoietic cytokine receptors.

A non-redundant signaling pathway distinguishing OSMR from the other receptors in the gp130 family of cytokines is recruitment of the adapter protein Shc1 (Src homology and collagen 1) to Tyr^861^ (26, 27). Shc proteins are phosphotyrosine adapters which link activated transmembrane receptors to downstream signaling cascades (28). Four members of this family have been described, designated Shc1, Shc2, Shc3 and Shc4. Three isoforms of Shc1 protein generated by differential promoter usage (p66) or alternative translational initiation (p46, p52) have been discovered. Shc1 contains both phosphotyrosine binding domains (PTB) and SH2 domains and is able to recruit the Ras/Raf/MAPK adapter Grb2 to the SH2 domain. Phosphorylation of the OSMR on Tyr^861^ allows binding of activated Shc1 to the OSMR, recruitment of Grb2 and subsequent induction of a Ras-dependent kinase cascade, which results in activation of MAPK (26). This is different from the LIF-induced activation of MAPK, where recruitment of SHP-2 to the gp130 subunit in the LIFR mediates activation of MAPK (2). Since OSMR lacks the recruitment motif for SHP-2, activation of Shc1 substitutes for SHP-2 mediated activation of the MAPK caused by the closely related LIFR, but the functional relevance of OSMR-Shc1 in bone has not been investigated. Interestingly, activation of Shc1 has also recently been shown to potentiate STAT3 phosphorylation in breast cancer cells (29), but a role for the OSMR-Shc1-STAT3 axis in osteoblasts has not been assessed.

The aim of the present study was to investigate the importance of the Shc1-STAT3 signaling pathway in OSM-induced RANKL formation in osteoblasts and subsequent osteoclast formation.

## Materials and Methods

### Materials

Recombinant mouse LIF, mouse OSM, bone morphogenetic protein-2 (BMP-2), macrophage colony-stimulating factor (M-CSF), RANKL (amino acids 158–316; cat. no. 462-TEC) and the ELISA kits for mouse RANKL and mouse OPG were purchased from R&D Systems, Abingdon, UK; bacterial collagenase type I from Worthington Biochemical Corp., Lakewood, NJ, USA; α-MEM, FBS, L-glutamine, and oligonucleotide primers from Invitrogen, Stockholm, Sweden; RNAqueous®-4PCR RNA isolation kit from Ambion, Inc., Austin TX, USA; 1st strand cDNA synthesis Kit and PCR Core Kit from Roche, Mannheim, Germany; DYEnamic ET terminator cycle sequencing kit from GE Healthcare, Uppsala, Sweden; QIAquick PCR Purification kit was from Qiagen Ltd., Crawley, West Sussex, England; TaqMan Universal PCR Master Mix and TaqMan probes from Applied Biosystems, Foster City, CA, USA; all primary and secondary antibodies used are specified in Supporting Information Table I; anti-IgG-HRP secondary antibodies used for Western blot were from Santa Cruz Biotechnology, Inc., Santa Cruz, CA, USA; culture dishes and multi-well plates from Costar, Cambridge, MA, USA, or Nunc International Corp., Naperville, IL, USA. Indomethacin was kindly supplied by Merck, Sharp & Dohme, Haarlem, the Netherlands; the mouse bone marrow stromal cell line ST-2 from Riken BRC Cell Bank (www.brc.riken.go.jp).

### Animals

CsA mice from the inbred colony at Umeå University, Swiss mice from the School of Dentistry at Araraquara and C57Bl/6 mice from the University of Gothenburg were used for isolation of calvarial osteoblasts, bone marrow cells or calvarial bone explants. The Institutional Animal Care and Ethics Committees at Umeå University, at the School of Dentistry, Araraquara and at the University of Gothenburg approved all experimental studies. The observation that OSM is a more robust stimulator than LIF of *Tnfsf11* (encoding RANKL) mRNA expression was made in cells from all three genotypes; OSM-induced phosphorylation of Shc1 was assessed in cells from CsA and Swiss mice and found to activate Shc1 in both strains.

### Bone Resorption Bioassay

Bone resorption was assessed in organ culture of parietal bones from 6 to 7 days-old mice by analyzing the release of ^45^Ca from prelabelled bones as previously described (30, 31). Release of isotope was expressed as the percentage release of the initial amount of isotope (calculated as the sum of radioactivity in medium and bone after culture). The data were recalculated and the results expressed as percent of control that was set at 100%, which allowed for accumulation of data from several experiments.

### Isolation and Culture of Mouse Calvarial Osteoblasts

Bone cells were isolated from calvariae harvested from 2 to 5 days-old mice using bacterial collagenase in the modified time sequential enzyme-digestion technique (32). Cells from populations 6 to 10, showing an osteoblastic phenotype as assessed by their cyclic AMP-responsiveness to PTH, expression of alkaline phosphatase, osteocalcin and bone sialoprotein, as well as the capacity to form mineralized bone noduli (data not shown), were used. The cells were seeded in culture flasks containing α-MEM supplemented with 10% FBS, L-glutamine and antibiotics at 37°C in humidified air containing 5% CO_2_. After 4 days, the cells were sub-cultured in culture dishes or multi-well plates.

### Osteoclast Differentiation in Bone Marrow Cell Cultures

Bone marrow cells (BMC) were flushed from femur and tibiae from 6 week-old mice and seeded in 48 multi-well plates containing α-MEM/10% FBS and incubated overnight. The cells were then cultured in the same medium with or without test substances for 7–9 days. Cells staining positive for tartrate-resistant acid phosphatase (TRAP) and containing three or more nuclei were considered osteoclasts and the number of TRAP-positive multinucleated osteoclasts (TRAP^+^ MuOCL) was counted.

### Osteoclast Differentiation in Bone Marrow Macrophage Cultures

Bone marrow cells from 6 to 12 weeks old mice were isolated and incubated in the presence of M-CSF (30 ng/mL) for 3 days in culture dishes, to which stromal cells and lymphoid cells cannot adhere, as previously described (33, 34). The cells adherent to the bottom of the dishes are devoid of phenotypic markers for stromal cells, T- and B-cells, express CD115/c-Fms (75%) and CD11b/Mac-1 (100%), and were used as bone marrow macrophages (BMM). The BMM cells were seeded in 96 multi-well plates and then incubated in M-CSF (30 ng/mL) or M-CSF + RANKL (30 ng/mL + 4 ng/mL) with or without LIF (100 ng/mL) or OSM (100 ng/mL). Cells staining positive for TRAP and containing three or more nuclei were considered osteoclasts and the number of TRAP^+^ MuOCL was counted.

### Stromal Cells

The ST-2 cells were seeded in multi-well plates and incubated in α-MEM/10% FBS overnight. Following incubation, medium with and without test substances was added and the cells incubated for 24 h for subsequent gene expression analysis.

### Gene Silencing in Osteoblasts Using Small Interfering RNA

Calvarial osteoblasts were seeded in multi-well plates with α-MEM supplemented with 10% FBS and antibiotics. For co-culture and gene expression experiments, 10^3^ cells were seeded per well in 96-well plates, while for protein extraction, 5 × 10^4^ cells/well were seeded in 12-well plates. After overnight attachment, silencing of *Osmr, Lifr, Il6st, Shc1* or *Stat3* was performed using Lipofectamine RNAiMAX and 30 nM of the appropriate siRNAs listed in Supporting Information Table II. Cells treated with a scrambled (siSCR) sequence served as controls. Forty-eight hours after the first silencing, the protocol was repeated. Twenty-four hours after the second silencing, the cells were incubated in medium containing either vehicle, LIF or OSM. At the end of cultures, RNA or protein was extracted. In some experiments, the osteoblasts were co-cultured with BMMs.

### Osteoblast and Bone Marrow Macrophage Co-cultures

Following silencing of *Shc1* or *Stat3* in osteoblasts, 2x10^4^ BMMs were added to each well in 96-well plates containing osteoblasts. The co-cultures were exposed to vehicle or OSM (100 ng/mL) and 3 days later, the cells were fixed with PBS-buffered 4% paraformaldehyde and stained for TRAP. The number of TRAP^+^ MuOCL was counted in each well.

### RNA Isolation and First-Strand cDNA Synthesis

Total RNA was extracted using commercially available RNA isolation kits (Ambion or Qiagen) by following the manufacturer's protocol. For quantitative real-time polymerase chain reactions, RNA was extracted from a single cell culture well, or from individual bones. For semi-quantitative polymerase chain reactions, RNA extracted from three wells was pooled per treatment group. RNA was reverse transcribed into single-stranded cDNA with a commercially available cDNA synthesis kit using.

### Semi-quantitative Polymerase Chain Reaction

Polymerase chain reaction analyses were performed using a standard protocol. The reaction conditions were: denaturing at 94°C for 2 min, annealing for 40 s, and elongation at 72°C for 60 s; in subsequent cycles denaturing was performed at 94°C for 40 s. Reaction conditions for OPG and RANKL were as follows: denaturation at 94°C for 35 s, annealing at 65°C for 35 s, and elongation at 72°C for 60 s for 10 cycles. In subsequent cycles, the primer annealing temperature was decreased stepwise by 5°C every 5 cycles from 65 to 45°C. The primer sequences (forward and reverse, given in the 5′-3′ orientation), expected fragment lengths and annealing temperatures used in PCR are listed in Supporting Information Table III. The expressions of the target genes were compared at the logarithmic phase of the PCR reaction. No amplification was detected in samples where the RT reaction had been omitted (data not shown). The PCR products were electrophoretically size fractionated in 1.5% agarose gel and visualized using ethidium bromide. The identity of the PCR products was confirmed using a DYEnamic ET terminator cycle sequencing kit with sequences analyzed on an ABI 377 XL DNA Sequencer (PE Applied Biosystems, Foster City, CA).

### Quantitative Real-Time Polymerase Chain Reaction

Quantitative real-time PCR analysis was performed using TaqMan kinetics. In each reaction, cDNA was amplified using a TaqMan Universal PCR Master Mix kit, 300 nmol/L of each primer and 100 nmol/L of probe on an ABI Prism 7900 HT Sequence Detection System (Applied Biosystems, Foster City, CA, USA) or predesigned Taqman Assays and Taqman Fast Advance Master Mix on a StepOnePlus Real-Time PCR system. The primers and probes used are listed in Supporting Information Tables III, IV. Gapdh (for BMM) or β-actin or 36B4 (for BMC and calvarial osteoblasts) were used as internal standard to correct for differences in starting mRNA concentrations.

### Protein Analyses of RANKL and OPG

The protein levels of RANKL and OPG in calvarial bones were analyzed using commercially available ELISA kits. Calvarial cells were lysed using 0.2% Triton X-100 and the extracted samples were analyzed by following the manufacturer's protocol.

### Preparation of Total Cell Lysates

Calvarial osteoblasts were seeded in 60 cm^2^ dishes at a density of 2 × 10^4^ cells/cm^2^. After 3 days of culture with one media change, the cells were incubated in the absence (control) or presence of test substances for different time periods. Following incubation, the cells were washed twice in PBS before addition of lysis buffer (1% Igepal CA-630, 0.1% SDS, 2 mM EDTA, 50 mM NaF, 0.1 mg/mL PMSF, 10 μg/mL leupeptin, 10 μg/mL pepstatin A, in PBS). The dishes were kept on ice for 15 min followed by scraping and collection of cell lysates. Before use in Western blot, cell lysates were concentrated using Microcon centrifugal filter devices according to manufacturer's recommendations. Protein concentration of the cell lysates was measured using the BCA method with bovine albumin as standard.

### Western Blot Analysis

For Western blot analysis, cell lysates pooled from three culture dishes were mixed with sample buffer (200 mM Tris-HCl, pH 6.7, 20% glycerol, 10% β-mercaptoethanol, 5% SDS, 0.01% Pyronin Y) and boiled for 3 min. Protein samples were then loaded on 4–12% Tris-HCl polyacrylamide gels and electrophoresis was performed according to the Laemmli method. Electrophoretically separated proteins were then blotted onto a PVDF membrane which was blocked (5% BSA in TBS) overnight. For detection, the membrane was incubated with primary antibody overnight in 1% BSA/PBS in dilutions specified in Supporting Information Table I. Three times 10 min of wash in TBS with 0.05% Tween-20 (TBST) was followed by incubation with HRP-conjugated secondary antibody (1:5,000 in 1% BSA, 0.05% Tween-20 in TBS) for 60 min at room temperature. Finally, the membrane was washed extensively with TBST and TBS followed by development using a chemiluminescence detection kit according to manufacturer's protocol.

### Preparation of Nuclear Extracts

Calvarial osteoblasts were plated at a density of 2 x 10^4^ cells/cm^2^ in culture dishes (60 cm^2^) containing α-MEM with 10% FBS, L-glutamine and antibiotics. After 4 days with one media change, the cells were incubated in the absence (control) or presence of test substances for 30 min. Following incubation, the cells were washed with ice cold PBS and scraped. Cell suspensions from two culture dishes were pooled and centrifuged briefly and pelleted cells homogenized in lysis buffer A (10 mM HEPES, pH 7.9, 0.1 mM EDTA, 10 mM KCl, 625 μg/mL spermidine, 625 μg/mL spermine, 0.5 mM DTT, 0.5 mM PMSF, 1 μg/mL leupeptin, 1 μg/mL pepstatin A). After 15 min on ice, Igepal CA-630 was added to a final concentration of 0.5%. The nuclei were collected by centrifugation at 12 000 x g for 2 min, and pelleted nuclei were lysed by incubation for 30 min on ice in lysis buffer B (20 mM HEPES, pH 7.9, 0.2 mM EDTA, 0.42 M NaCl, 25% glycerol, 625 μg/mL spermidine, 625 μg/mL spermine, 0.5 mM DTT, 0.5 mM PMSF, 1 μg/mL leupeptin, 1 μg/mL pepstatin A). Supernatants were collected by centrifugation at 16 000 x g for 10 min. The protein concentration of the samples was determined by the Bradford method and aliquots were stored at −80°C until use in electrophoretic mobility shift assays (EMSAs).

### EMSA

Consensus oligonucleotides including an AP-1 site (CGCTTGATGACTCAGCCGGAA) and a κB site (AGTTGAGGGGACTTTCCCAGGC) were end-labeled with [γ-^32^P] ATP using T4 kinase according to manufacturer's instructions. Mutated forms of the AP-1 (CGCTTGATGACT) and NF-κB (AGTTGAGG) oligonucleotides were used in competition studies. Annealing of complementary strands of both labeled and unlabelled oligonucleotides was performed before used in electrophoretic mobility shift assay (EMSA). Reaction mixtures containing 8 μg of nuclear extract, 0.5–1 ng of probe (50 000 cpm), 4 μg poly(dI-dC)•poly(dI-dC), 20 nM DTT, and reaction buffer (50 mM Tris-HCl, pH 7.5, 0.25 M NaCl, 5 mM EDTA, 25% glycerol) were incubated at room temperature for 30 min. In antibody supershifts and competition studies, 2 mg/mL of antibody, or 50- or 100-fold excess of unlabelled probe, was pre-incubated with reaction mixture without probe for 30 min before addition of ^32^P-labeled probe. After incubation for 30 min at room temperature, samples were loaded onto a non-denaturing polyacrylamide gel and electrophoresed, followed by drying of the gel and autoradiography.

### Statistical Analysis

Statistical significance was determined by ANOVA using Levene's homogeneity test and Dunnett's 2-sided, Dunnett's T3 or Tukey's post hoc test. When comparing two groups, non-parametric Mann-Whitney U test, or two-sided Student's *t*-test was used, where applicable. A *P* < 0.05 was considered statistically significant. Statistical significance is presented as follows, ^*^P < 0.05, ^**^P < 0.01, and ^***^P < 0.001. Data are expressed as mean ± SEM. Numerical values are expressed as percent of unstimulated control, with controls presented as 100% or 1-fold, if not otherwise stated. We have used four wells or six bones (^45^Ca release) per treatment group and then calculated a mean value and standard variation (SEM) for each group, which have been used for statistical analyses and to create the figures. The experiments have then been repeated using the same design and calculations 2–3 times with similar results.

## Results

### OSM, in Contrast to LIF, Robustly Activates ERK and the AP-1 Complex in Calvarial Osteoblasts

**Figure 1:**
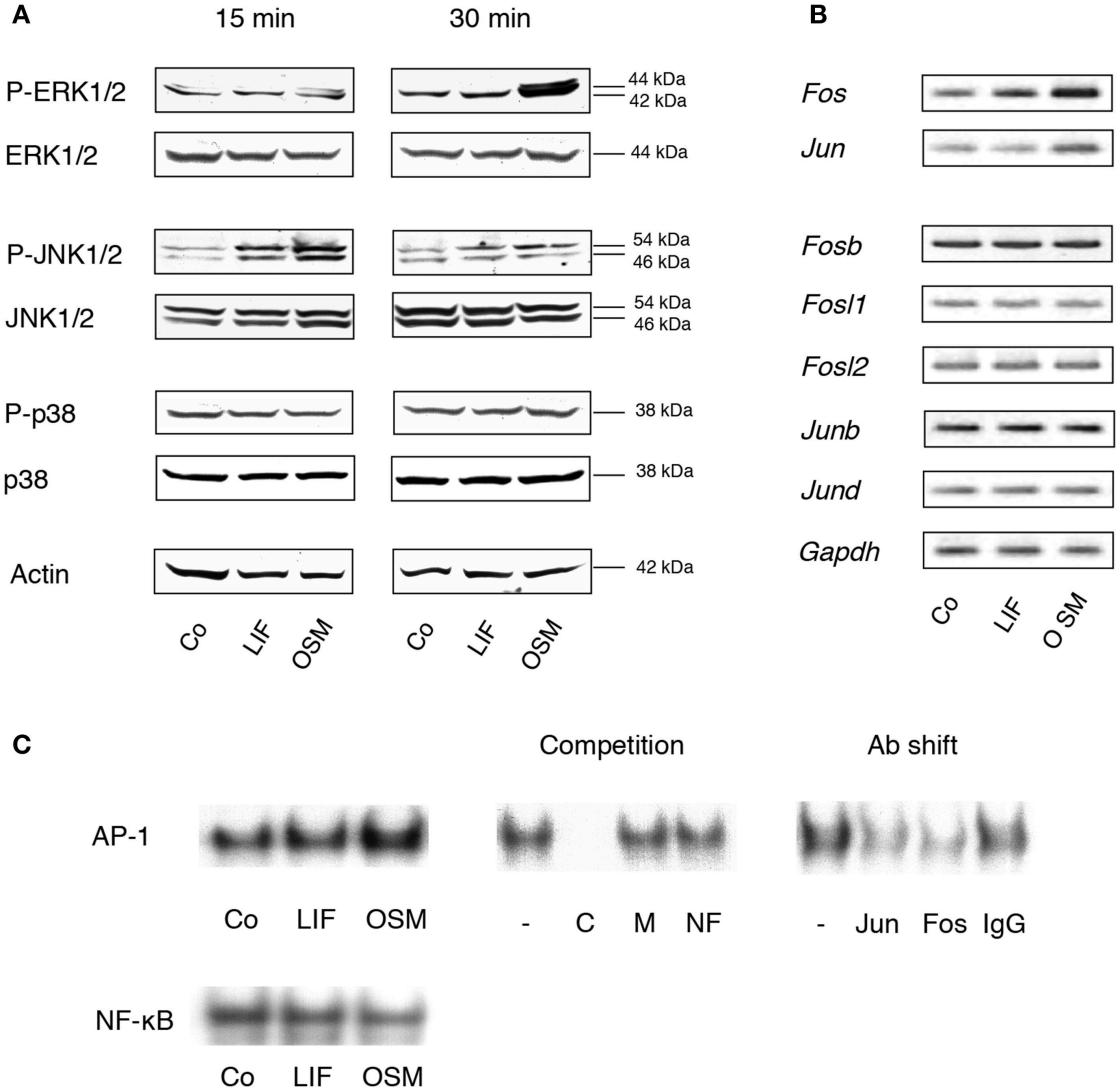
MAP kinases and the AP-1 complex are differentially activated by OSM and LIF. **(A)** Mouse calvarial osteoblasts were cultured in the absence (Co) or presence of LIF or OSM (both at 100 ng/mL) for 15 and 30 min followed by cell lysis and Western blot analysis of total, as well as phosphorylated, ERK, JNK and p38. Actin served as the internal control for protein loading. **(B)** Semi-quantitative PCR analysis of AP-1 subunit mRNA expression after incubation without (Co) or with LIF or OSM (both at 100 ng/mL) for 48 h. *Gapdh* served as loading control. **(C)** EMSA analysis of nuclear extracts from cells incubated for 30 min in the absence (Co) or the presence of LIF or OSM (both at 100 ng/mL). Left upper panel, EMSA for nuclear extracts incubated with AP-1 consensus probe. Middle upper panel, competition studies on nuclear extracts from OSM-stimulated cells. From left: No competitor (–), homologous unlabelled (cold) AP-1 consensus probe (C), mutated AP-1 consensus probe (M), non-homologous probe (NF-κB; NF). Right upper panel, supershifts using antibodies against c-Jun (Jun), c-Fos (Fos), and unspecific IgG (IgG) of nuclear extracts from OSM-stimulated cells. Lower panel, EMSA for nuclear extracts incubated with NF-κB consensus probe.

Stimulation of calvarial osteoblasts with OSM (100 ng/mL) resulted in a robust, time-dependent activation of the MAP kinase ERK, as assessed by phosphorylation of Tyr^204^ (Figure 1A). OSM treatment also resulted in increased phosphorylation of the MAP kinase JNK on Tyr^185^ and Thr^183^. In contrast, OSM did not stimulate phosphorylation of the p38 MAPK on Tyr^182^. Treatment of the osteoblasts with LIF (100 ng/mL) caused a weak, rapidly transient activation of JNK, but did not affect phosphorylation of ERK or p38. Neither OSM nor LIF had any effect on total protein levels of p38, JNK or ERK (Figure 1A).

Activation of AP-1 is a consequence of MAPK activation and we, therefore, next studied the effects of OSM on transcriptional control of AP-1 subunits and DNA binding of AP-1, as assessed by semi-quantitative RT-PCR and EMSA, respectively.

OSM (100 ng/mL) increased the mRNA expression in mouse calvarial osteoblasts of *Fos* and *Jun* after 48 h treatment (Figure 1B), whereas *Fosb, Fosl1, Fosl2, Junb*, and *Jund* mRNA expressions were unaffected (Figure 1B). In contrast, LIF caused a marginal increase of *Fos* mRNA but did not affect the expression of the other AP-1 subunits (Figure 1B).

Incubation of calvarial osteoblasts with OSM for 30 min resulted in enhanced DNA binding of AP-1 as assessed by EMSA, whereas LIF had no effect (Figure 1C, left top panel). The binding specificity was evident by the complete displacement with a 50-fold excess of unlabelled (cold) homologous oligonucleotide (C), whereas a mutated homologous oligonucleotide (M) and a non-homologous oligonucleotide (NF-κB; NF) had no effect (Figure 1C, middle top panel). The antibody shift experiment demonstrated the involvement of both c-Jun (Jun) and c-Fos (Fos) in the AP-1 complex activated by OSM (Figure 1C, right top panel). In contrast to the enhanced AP-1 DNA binding activity by OSM, treatment with either OSM or LIF did not result in an effect on NF-κB DNA binding activity (Figure 1C, lower panel).

These observations demonstrated clear differences in signaling events downstream the OSM and LIF receptors in primary mouse calvarial osteoblasts, which are in line with observations made in the human osteoblastic MG-63 cell line showing a clear activation of ERK by OSM, but a weaker by LIF (35).

### OSM Is a More Robust Activator Than LIF of STAT3 mRNA Expression and Phosphorylation

**Figure 2:**
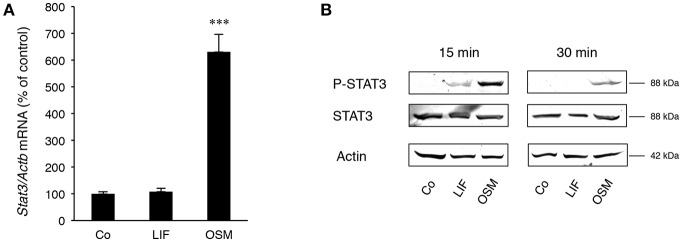
STAT3 is differentially expressed and activated by OSM and LIF. Mouse calvarial osteoblasts were cultured in the absence (Co) or presence of LIF or OSM (both at 100 ng/mL) for 48 h to assess *Stat3* mRNA expression by RT-qPCR **(A)** or for 15 and 30 min to assess phosphorylated and total STAT3 protein levels by Western Blot **(B)**. Values in **(A)** represent means for four wells and SEM is shown as vertical bars. Significant differences compared to untreated cells (Co) is defined as ^***^P < 0.001 analyzed by one-way ANOVA followed by Dunnet's multiple comparison test vs. Co.

Stimulation of calvarial osteoblasts with OSM (100 ng/mL) increased expression of *Stat3* mRNA, whereas no such effect was observed after stimulation with LIF (Figure 2A).

Western blot analyses indicated that phosphorylation of the transcription factor STAT3 on Tyr^705^, which is crucial for dimerization of STAT3 and subsequent DNA binding (36), was much greater after treatment with OSM than LIF (Figure 2B). No effect on total STAT3 protein by OSM or LIF was observed.

These findings show that OSM robustly activates STAT3, whereas LIF only causes a marginal activation of this transcription factor.

### Activation by OSM of the Adapter Protein Shc1 in Calvarial Osteoblasts Is Crucial for STAT3 and ERK Activation

Having observed the differences in MAPK and STAT3 activation in osteoblasts stimulated with OSM and LIF, and knowing that only OSM has been reported to activate the adapter protein Shc1 in several other cell types, we next investigated the role of Shc adapters for the robust activation by OSM of ERK and STAT3 in osteoblasts. The expression of Shc proteins in osteoblasts had not been examined previously and to determine the role of Shc, the expression pattern of Shc proteins in calvarial osteoblasts treated with either OSM or LIF was evaluated.

**Figure 3:**
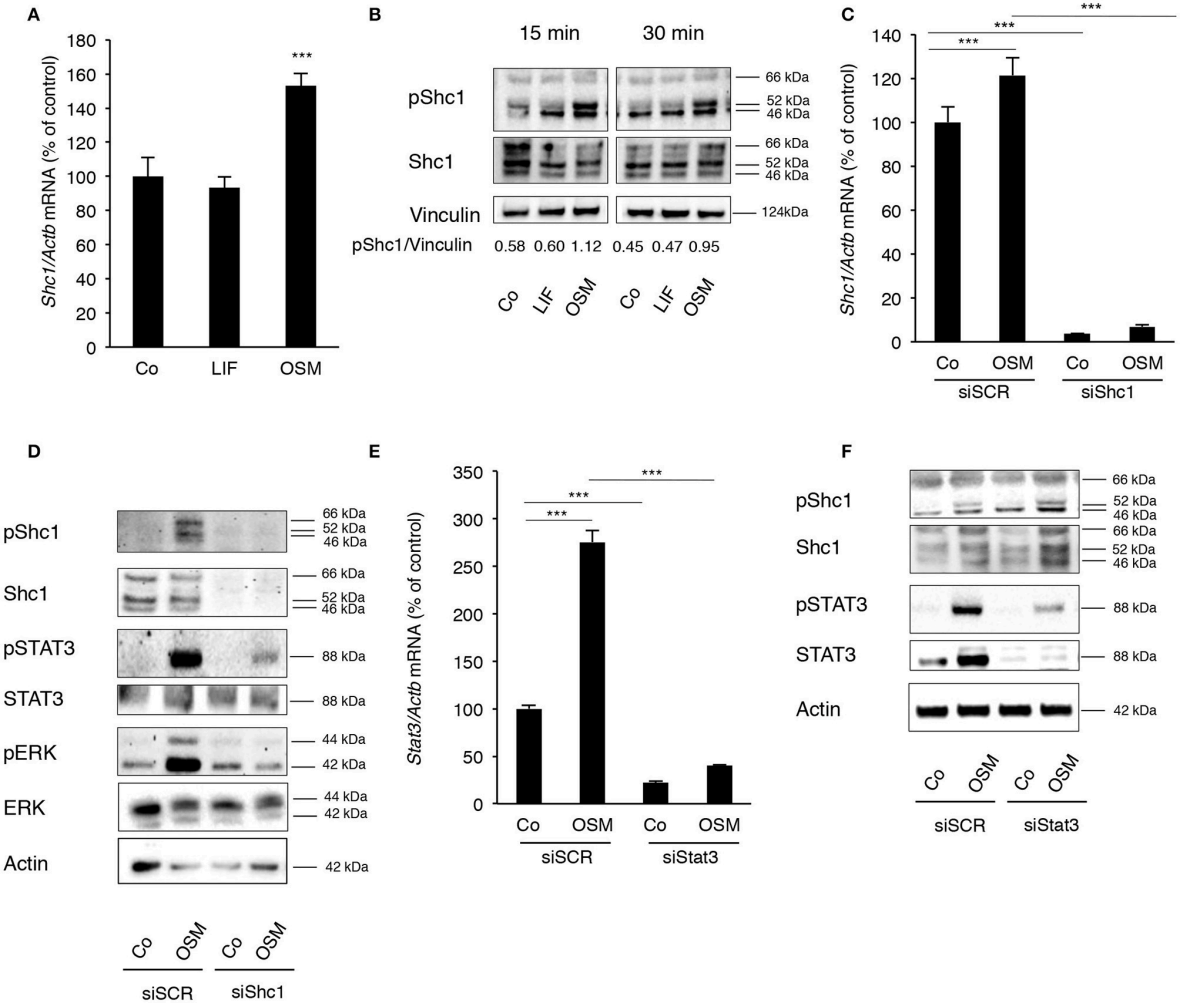
OSM stimulates activation of STAT3 and ERK by phosphorylating the adapter protein Shc1. **(A)** Mouse calvarial osteoblasts were cultured in the absence (Co) or presence of LIF or OSM (both at 100 ng/mL) for 24 h and RNA was extracted for RT-qPCR analysis of *Shc1*mRNA. **(B)** The cells were exposed to LIF or OSM at 100 ng/mL for 15 and 30 min followed by cell lysis and Western blot for phosphorylated Shc1; the bands were quantified and normalized by vinculin. **(C)** Calvarial osteoblasts had the *Shc1* gene silenced by small interfering RNA and were exposed to LIF or OSM (both at 100 ng/mL) for 24 h before RNA extraction and analysis of *Shc1* mRNA expression by RT-qPCR; a scrambled sequence (siSCR) served as control. **(D)** Osteoblasts that had the *Shc1*gene knocked-down by siRNA and control cells exposed to scrambled sequence (siSCR) were exposed to OSM at 100 ng/mL or vehicle for 15 min and proteins were extracted for analysis of the phosphorylation of Shc1, STAT3 and ERK. **(E)** The *Stat3* gene was silenced in calvarial osteoblasts, which were subsequently exposed to OSM at 100 ng/mL or vehicle (Co) for 24 h before analysis of *Stat3* gene expression by RT-qPCR. **(F)** The effect of *Stat3* silencing on phosphorylation of STAT3 and Shc1 was analyzed by Western blot in osteoblasts treated with OSM for 15 min. **(A,C,E)** Values represent means for four wells and SEM is shown as vertical bars. **(A)** Significant differences compared to untreated cells (Co) at each time point are defined as ^***^P < 0.001; analyzed by one-way ANOVA followed by Dunnet's post *hoc-test*. **(C,E)** Significant differences are indicated by horizontal lines where ^***^P < 0.001 two-way ANOVA followed by Tukey post *hoc-test*. The difference in OSM-induced response with and without silencing analyzed by two-way ANOVA was statistically significant [interaction *P*-value in **C** (*P* < 0.005) and in **E** (*P* < 0.0001)]. OSM had no statistically significant effect (*P* > 0.05) on *Shc1* or *Stat3* mRNA expression in cells which had been silenced for *Shc1* or *Stat3*, respectively **(C,E)**.

Calvarial osteoblasts expressed *Shc1* (Figure 3A), but not *Shc2, Shc3* or *Shc4* mRNA (data not shown). Interestingly, *Shc1* mRNA was upregulated by OSM (Figure 3A). All three isoforms of Shc1 protein, p46, p52, and p66, were expressed by the osteoblasts (Figure 3B) and activation by OSM stimulated the phosphorylation of Shc1 (Figure 3B). OSM activated the phosphorylation of the p52 isoform in all experiments, but phosphorylation of the other isoforms was also observed in some experiments. In contrast to OSM, LIF did not affect *Shc1* mRNA expression or phosphorylation of Shc1 protein (Figures 3A,B).

The importance of Shc1 for OSM-induced STAT3 and ERK activation was then determined by silencing *Shc1* expression in the osteoblasts. The siRNA used decreased the mRNA expression of *Shc1* by 97% in control cells, as well as caused a significant decrease of *Shc1* mRNA in OSM-treated cells (Figure 3C). The silencing subsequently resulted in effective reductions of Shc1 protein expression and phosphorylation (Figure 3D). Decreased Shc1 activation substantially decreased OSM-induced phosphorylation of STAT3 and ERK, without affecting total protein levels of STAT3 and ERK (Figure 3D).

After observing a robust activation of STAT3 by OSM, the role of STAT3 phosphorylation in the OSM-induced activation of Shc1 was evaluated. Treatment of osteoblasts with OSM (100 ng/mL) resulted in a 2.8-fold increase of *Stat3* mRNA expression (Figure 3E). Silencing of *Stat3* expression decreased the mRNA expression of *Stat3* in untreated control cells by 80% and significantly decreased OSM-induced upregulation of *Stat3* mRNA (Figure 3E). Silencing of *Stat3* resulted in a decrease in STAT3 protein expression and OSM-induced STAT3 phophorylation, as expected, but did not affect phosphorylation of Shc1 (Figure 3F). The fact that silencing of *Shc1* resulted in impaired phosphorylation of STAT3 induced by OSM, whereas silencing of *Stat3* did not decrease the OSM-induced activation of Shc1 (Figure 3F), indicate that Shc1 activation is upstream of STAT3.

### Activation of STAT3 and the Adapter Protein Shc1 by OSM Is Crucial for Stimulation of RANKL Expression and Osteoclastogenesis

**Figure 4:**
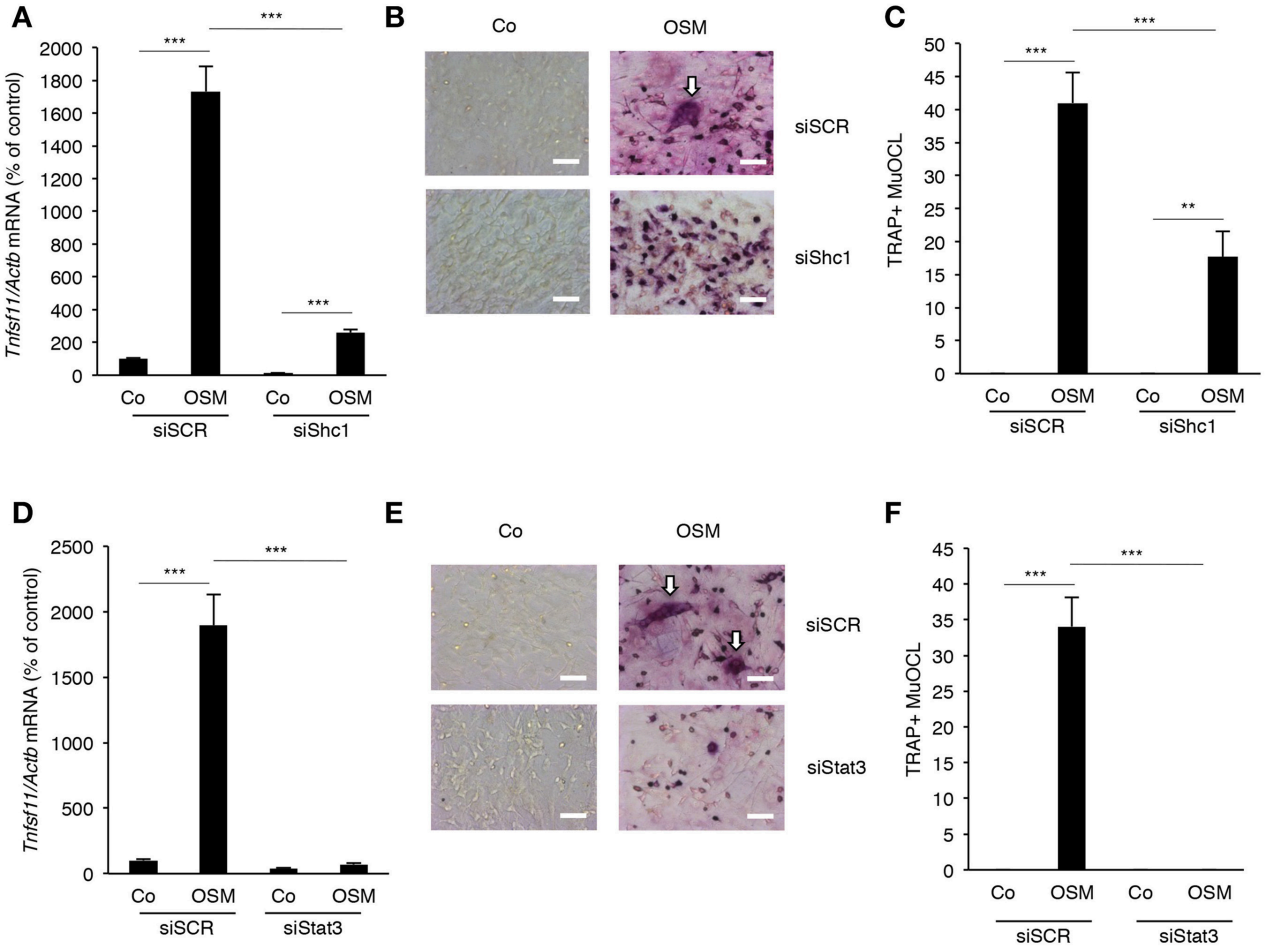
Expression of RANKL in osteoblasts and osteoclastogenesis in co-cultures of bone marrow macrophages and calvarial osteoblasts induced by OSM are dependent on Shc1 and STAT3. Osteoblasts in which the *Shc1* gene **(A)** or the *Stat3* gene **(D)** was knocked-down by siRNA were treated with OSM at 100 ng/mL for 24 h before analysis of *Tnfsf11* gene expression. Osteoblasts transfected with scrambled RNA (siSCR) were similarly treated and analyzed. Osteoblasts in which *Shc1* **(B,C)** or *Stat3* **(E,F)** was knocked down (or siSCR as control) were exposed to OSM (100 ng/mL) or vehicle and co-cultured with BMMs for 3 days before TRAP staining and counting of TRAP^+^MuOCL **(A,C,D,F)**. Values represent means for four wells and SEM is shown as vertical bars. Significant differences are indicated by horizontal lines where ^**^P < 0.01; ^***^P < 0.001; analyzed by two-way ANOVA followed by Tukey post *hoc-test*. The difference in OSM-induced response with and without silencing analyzed by two-way ANOVA was statistically significant [interaction *P*-value in **A** (*P* < 0.0001), **C** (*P* < 0.005), **D** (*P* < 0.0001) and **F** (*P* < 0.0001)]. Scale bar in **(B,E)** is 50 μm.

Next, the importance of the Shc1-ERK and JAK-STAT3 pathways for stimulation of RANKL expression by OSM was evaluated. Silencing of *Shc1* significantly decreased the OSM-induced mRNA expression of *Tnfsf11* (encoding RANKL) (Figure 4A). Importantly, silencing of *Shc1* expression in osteoblasts decreased OSM-induced osteoclast formation in co-cultures of calvarial osteoblasts and BMM (Figures 4B,C). Silencing of *Stat3* also resulted in substantially decreased expression of OSM-induced T*nfsf11* mRNA expression (Figure 4D), and totally prevented formation of osteoclasts in OSM-stimulated co-cultures of calvarial osteoblasts and BMM (Figures 4E,F).

### OSM Is a More Robust Stimulator of ^45^Ca Release and Expression of RANKL in Mouse Calvarial Bones and Calvarial Osteoblasts

Having observed that OSM but not LIF receptors activate Shc1-dependent signaling, we next assessed the importance of this difference for bone resorption, RANKL production and osteoclastogenesis.

**Figure 5:**
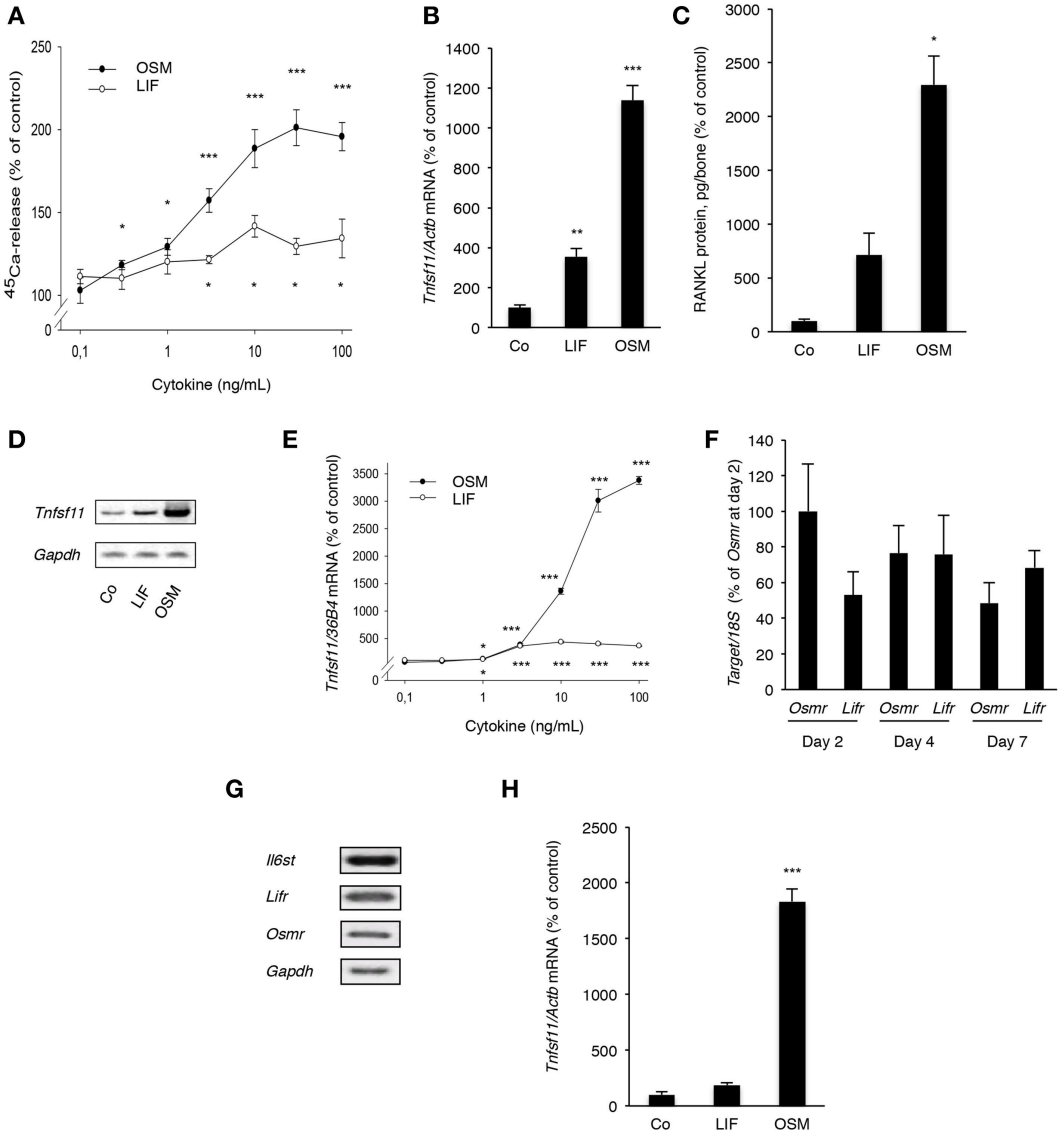
OSM and, to a lesser extent, LIF stimulate bone resorption and RANKL production in calvarial bones, calvarial osteoblasts and stromal cells. **(A)** Mouse calvarial bones were cultured in the absence or the presence of LIF or OSM (both 0.1–100 ng/mL) and bone resorption was assessed by ^45^Ca release after a 5-day culture period. **(B)** RT-qPCR was performed using mRNA extracted from calvarial bones treated with either LIF or OSM (both at 100 ng/mL) for 24 h to assess the expression of *Tnfsf11*. **(C)** Protein expression of RANKL after 48 h was also analyzed in calvarial bone treated with LIF or OSM (both at 100 ng/L). **(D)** Mouse calvarial osteoblasts were incubated in the absence (Co) or the presence of LIF (100 ng/mL) or OSM (100 ng/mL) for 48 h and expression of *Tnfsf11* was analyzed by semi-quantitative RT-PCR. **(E)** The expression of *Tnfsf11* mRNA in calvarial osteoblasts stimulated by LIF and OSM a different concentrations (0.1-100 ng/mL) was performed using quantitative RT-PCR. **(F)** The mRNA expression of *Osmr* and *Lifr* in osteoblasts was compared at three different time points. **(G)** The receptor components *Il6st, Lifr* and *Osmr* are expressed in ST-2 stromal cells as assessed by RT-PCR. **(H)** The mRNA expression of *Tnfsf11* in ST-2 cells cultured without (Co) or with LIF or OSM (both at 100 ng/mL) for 48 h was analyzed. Values represent means for six bones (calvarial bones) or four wells (cell culture experiments) and SEM is shown as vertical bars. ^*^, ^**^, and ^***^, indicate significant difference compared to untreated (Co) cells, ^*^P < 0.05, ^**^P < 0.01, and ^***^P < 0.001, respectively. Statistical significance was determined by ANOVA using Levene's homogeneity test followed by Dunnett's T3 *post-hoc* tests vs. Co. In **(F)**, Tukey *post-hoc* test was used to compare all groups and no statistical difference was observed.

Both OSM and LIF stimulated bone resorption, as assessed by ^45^Ca release from neonatal mouse calvarial bone, in a concentration-dependent manner with OSM being a substantially more effective and potent stimulator than LIF (Figure 5A). The effects seen were statistically significant at and above 0.3 ng/mL (10 nM) OSM and 3 ng/mL (140 nM) LIF.

Quantitative PCR analysis revealed that OSM, and to a lesser extent LIF, increased mRNA expression of *Tnfsf11* in the calvarial bones (Figure 5B). This was in line with the finding that the protein level of RANKL in the mouse calvarial bones was increased 23-fold by OSM and 7-fold by LIF, respectively (Figure 5C).

Calvarial osteoblasts responded to LIF and OSM (both at 100 ng/mL) with enhanced *Tnfsf11* mRNA expression, with OSM clearly being the more effective stimulator (Figure 5D). Quantitative real-time PCR analysis showed that the difference in responsiveness between OSM and LIF could be observed over a wide range of concentrations with OSM treatment resulting in a more robust stimulatory effect on *Tnfsf11* mRNA expression (Figure 5E). The difference in response seemed not to be due to differences in receptor expression, as assessed by the mRNA expression of *Osmr* and *Lifr* (Figure 5F).

The stimulatory effect of OSM on *Tnfsf11* mRNA in the calvarial osteoblasts was dependent on the expression of *Il6st* (encoding gp130) and *Osmr*, but independent of *Lifr* expression, as demonstrated by silencing of the expression of these three receptor components in the calvarial osteoblasts (Supporting Information Figure S1). These findings are in agreement with previously reported observations that mouse OSM did not induce *Tnfsf11* mRNA in osteoblasts from *Osmr*^−/−^ mice (5).

The ST-2 stromal cells expressed mRNA for *Il6st, Lifr* and *Osmr* (Figure 5G) and responded to OSM with a robust 18-fold increase of *Tnfsf11* mRNA expression, whereas LIF did not cause any significant effect (Figure 5H).

### OSM but Not LIF Stimulates Osteoclast Formation and Expression of RANKL in Mouse Bone Marrow Cultures

**Figure 6:**
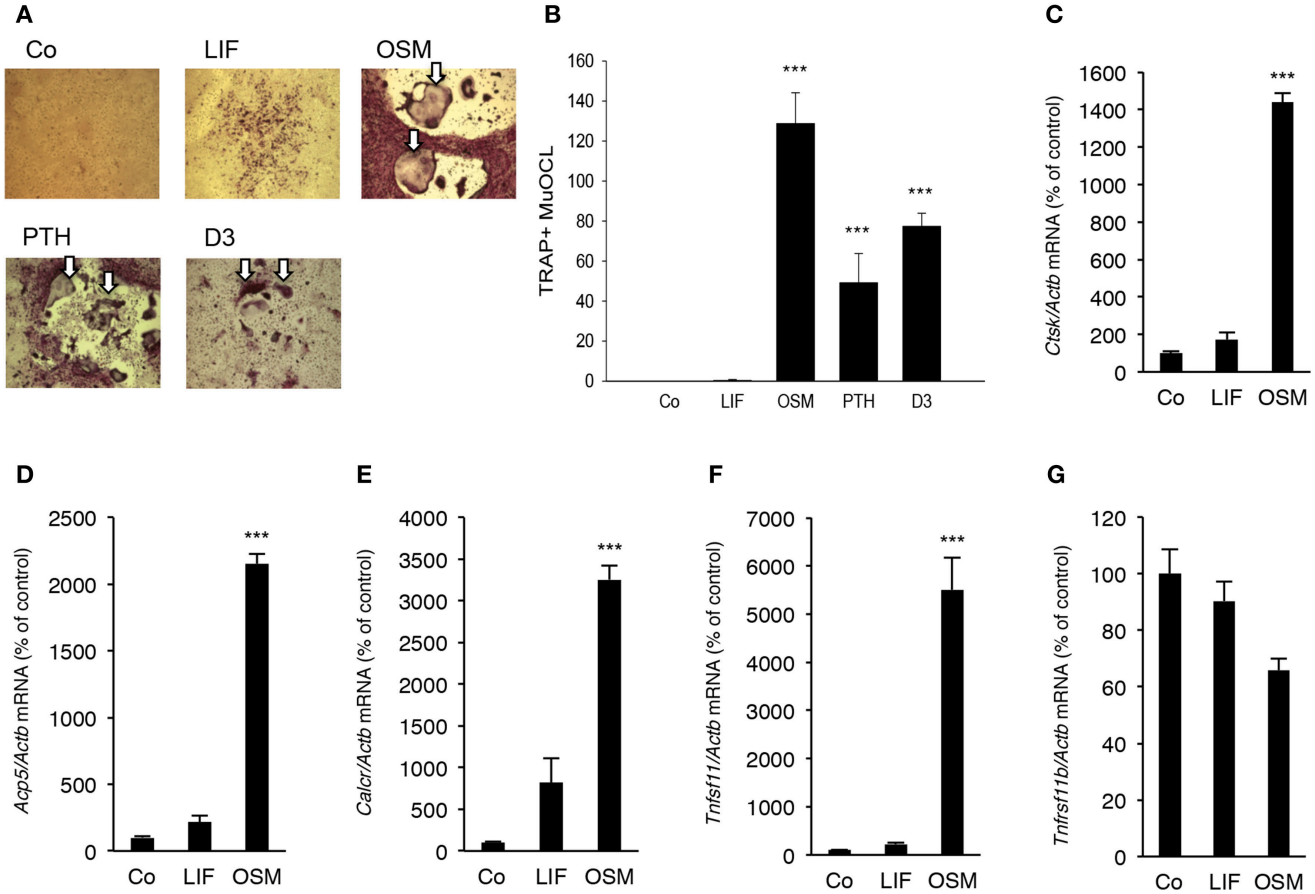
OSM, but not LIF, stimulates osteoclastogenesis in bone marrow cell cultures. Mouse bone marrow cells (BMC) were cultured in the absence (Co) or the presence of LIF, or OSM (both at 100 ng/mL), PTH or 1,25(OH)_2_-vitamin D3 (D3; both at 10^−8^ M) for 7 days before staining **(A)** and counting **(B)** of tartrate-resistant acid phosphatase (TRAP)-positive multinucleated osteoclasts (TRAP^+^MuOCL). Quantitative real-time PCR analysis of mRNA expression of cathepsin K (*Ctsk*, **C**), TRAP (*Acp5*, **D**), calcitonin receptor (*Calcr*, **E**) RANKL (*Tnfsf11*, **F**) and OPG (*Tnfsf11b*, **G**) in BMC cultured without (Co) or with LIF or OSM (both at 100 ng/mL) for 7 days. Values represent means for four wells and SEM is shown as vertical bars. ^***^, indicates significant difference compared to untreated (Co) cells, ^***^P < 0.001. Statistical significance was determined by ANOVA using Levene's homogeneity test followed by Dunnett's 2-sided **(B–F)** or Dunnett's T3 **(G)** *post-hoc* test.

Addition of OSM (100 ng/mL) for 7 days to mouse bone marrow cell (BMC) cultures significantly stimulated the formation of TRAP^+^ MuOCL (Figures 6A,B). The stimulation by OSM was more pronounced than stimulations caused by maximally effective concentrations of parathyroid hormone (PTH) and 1,25(OH)_2_-vitamin D3. In contrast, treatment with LIF (100 ng/mL) only resulted in formation of a few osteoclasts (Figures 6A,B).

It was also found that OSM (100 ng/mL) caused a 14–32 fold enhancement of the mRNA expression of the osteoclastic markers *Ctsk* (encoding cathepsin K), *Acp5* (encoding TRAP, tartrate-resistant acid phosphatase) and *Calcr* (encoding calcitonin receptor), whereas LIF (100 ng/mL) only caused a small 2–8 fold stimulation of the mRNA expression of these genes which was not statistically significant (Figures 6C–E).

OSM upregulated *Tnfsf11* mRNA in the BMC cultures 22-fold but did not affect *Tnfrsf11b* mRNA (encoding OPG) (Figures 6F,G). No significant effect of LIF on *Tnfsf11* or *Tnfrsf11b* mRNA was observed in the BMC cultures (Figures 6F,G).

### OSM and LIF Have No Direct Effect on Osteoclastogenesis

**Figure 7:**
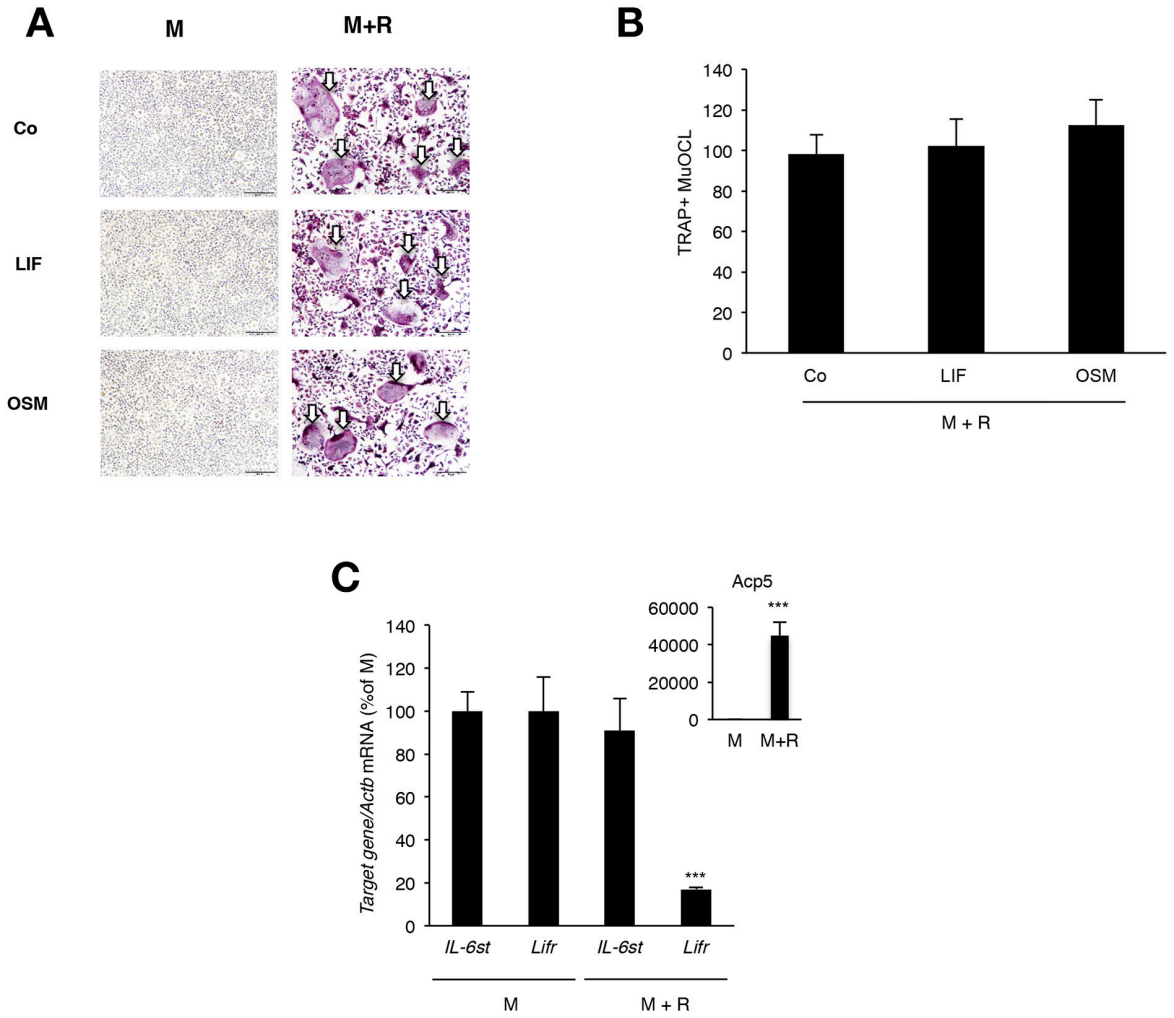
Lack of effect by OSM and LIF on osteoclast formation in bone marrow macrophage cultures. **(A,B)** BMMs were cultured in M-CSF (30 ng/mL) or differentiated to osteoclasts by addition of M-CSF + RANKL (30 and 4 ng/mL, respectively) in the presence or absence of LIF or OSM (both at 100 ng/mL) for 3 days before staining for tartarate-resistant acid phosphatase (TRAP)-positive multinucleated osteoclasts (TRAP^+^MuOCL), which then were counted. No osteoclasts were formed in the absence of RANKL. **(C)** Quantitative PCR analyses of *Il6st, Lifr*, and *Acp5* in BMM cultured for 72 h with M-CSF (30 ng/ml) or M-CSF+RANKL (30 and 4 ng/ml, respectively). Values represent means for four wells and SEM is shown as vertical bars. In **(C)** ^***^, indicates significant difference compared to cells treated with M-CSF (M), *P* < 0.001. In **(B)** Statistical significance was determined one-way ANOVA followed by Tukey's *post-hoc* test and in **(C)**, Student's *t*-test was used for comparison between M and M + R groups for each gene.

Analysis of BMM cultures revealed that neither LIF nor OSM stimulated formation of TRAP^+^ MuOCL in M-CSF treated BMM (Figure 7A). The cytokines also did not affect osteoclastogenesis when the formation of TRAP^+^ MuOCL in BMM was stimulated by M-CSF and RANKL (Figures 7A,B). In M-CSF- and M-CSF+RANKL-stimulated BMM, mRNA of *Il6st* (encoding gp130) and *Lifr* could be detected, but mRNA of *Osmr* was not detected (Figure 7C). RANKL-induced differentiation, as demonstrated by time-dependent upregulation of mRNA levels for *Acp5* (encoding TRAP), did not affect the expression of *Il6st* mRNA, but down-regulated *Lifr* mRNA expression (Figure 7C).

## Discussion

The present study shows that OSM is a more effective stimulator of bone resorption in mouse calvarial bones and a more potent stimulator of osteoclast differentiation and formation in mouse bone marrow cultures than is LIF. These effects were due to indirect effects mediated by osteoblasts/stromal cells rather than due to direct effects on osteoclast progenitor cells. Similarly, Tamura et al. found that LIF was a weaker stimulator of osteoclast formation than OSM in co-cultures of mouse osteoblasts and bone marrow cells (20). Other studies evaluating OSM and LIF have also noted a similar hierarchy of action for the two cytokines using mouse embryonic fibroblasts and rat hepatocytes (37), synovial fibroblasts (38, 39), NIH 3T3 fibroblasts and mouse lung fibroblasts (40). In the present study, we provide evidence that the difference in effects on osteoclastogenesis and bone resorption can be explained by recruitment of the adapter protein Shc1 to the OSMR. This recruitment results in a more robust activation of ERK/STAT3 signaling and expression of RANKL by OSM in comparison to LIFR-mediated signaling.

Heterodimerization of either OSMR:gp130 or LIFR:gp130 results in activation of both JAK/STAT and JAK/SHP-2/Grb2/Sos/Ras/Raf/MAPK signaling through docking of STATs and SHP-2 to different phosphorylated Tyr sites in the gp130 molecule (2, 6, 35, 41). The role of these pathways for bone mass has been assessed in “knock-in” mutant mice, one in which the C-terminal moiety of gp130 has been deleted (*gp130*^Δ*STATΔSTAT*^) to reduce STAT1/3 signaling, and another strain of mice in which a point mutation substituting Tyr^757^ (equivalent to Tyr^759^ in human gp130) with Phe^757^ (*gp130*^*Y*757*F*/*Y*757*F*^) blocks signaling through the SHP-2/MAPK pathway (42). In a parallel study, mice with a knock-in of gp130 carrying a substitution of Tyr^759^ with Phe^759^ (designated *gp130*^*F*759/*F*759^), which also results in defective SHP-2/MAPK signaling, have been used (43). These studies suggest that SHP-2/MAPK signaling, rather than STAT1/3 signaling, is important for basal bone remodeling and bone mass. However, the activation of downstream signaling pathways by specific cytokines in the gp130 family was not studied in these mice.

The fact that no difference in the mRNA expression of *Osmr* and *Lifr* in osteoblasts was observed indicate that the more robust stimulation of osteoclast formation by OSM is not likely to be due to differences in receptor numbers, but rather explained by differences in downstream signaling.

We found that OSM was a more robust stimulator than LIF of JNK (Tyr^185^/Thr^183^) and ERK (Tyr^204^) in mouse calvarial osteoblasts. This is in agreement with the study by Walker et al. reporting that OSM activates ERK more robustly than LIF in primary osteoblasts. Activation of the transcription factor AP-1 is an important downstream event following MAPK activation and experiments were conducted to determine if OSM affected DNA binding of AP-1 in calvarial osteoblasts (5). In agreement with the activation of ERK by OSM, it was determined with EMSA analysis that increased DNA binding of AP-1 occurred with OSM in calvarial osteoblasts, but not with LIF. Gelshift studies showed that the bound AP-1 heterodimer consisted of c-Fos and c-Jun subunits. In contrast to the increased DNA binding of AP-1 by OSM, EMSA analysis did not show any effect on the DNA binding of NF-κB by OSM or LIF, an observation that is in agreement with EMSA analysis in OSM-stimulated human peritoneal mesothelial cells (44).

Activation of JAK/STAT signaling is a well documented signaling event for cytokines in the IL-6 family. It has previously been reported that OSM and LIF can activate STAT1, STAT3, and STAT5 in osteoblasts, and that signaling through OSMR results in more robust activation of these transcription factors (5). In agreement with these observations, we found that OSM is a more robust activator of STAT3 than LIF, observations which also are in agreement with Itoh et al., who found that OSM caused a more robust and prolonged phosphorylation of STAT3 than LIF in osteoblasts from wild type mice (43). These findings likely explain why OSM more effectively than LIF enhanced the mRNA and protein expression of RANKL in calvarial bone, calvarial osteoblasts and bone marrow stromal cells. Similarly, O'Brien et al. have demonstrated that OSM-induced *Tnfsf11* mRNA in the UAMS-32 cell line was decreased by transfection with a dominant negative *Stat3* retrovirus (18) and investigators have showed that that silencing of *Stat3* impairs up-regulation of *Tnfsf11* mRNA by OSM in ST2 cells (45, 46). The important role of STAT3 for OSM-induced signaling in osteoblasts has also been demonstrated by the finding that OSM-induced activation of cyclin-dependent kinase inhibitor *p21*^*WAF*1, *CIP*1, *SD*11^ in the human osteosarcoma cell line MG-63 can be inhibited by transfection with a dominant negative *Stat3* plasmid (47). Furthermore, OSM promotes the binding of STAT3 and RNA polymerase II to 5′ enhancer regions in the *Tnfsf11* promoter shared by PTH and 1,25-dihydroxyvitamin-D3 (48) and to a more distal enhancer region shared by IL-6 (45).

An explanation why OSM is a more robust activator of JAK/STAT and MAPK signaling in the osteoblasts compared to LIF may be that phosphorylated Tyr^861^ in the OSMR acts as a binding site for an SH2 domain in the adapter protein Shc1, which is not recruited to the LIFR:gp130 complex (26). The Shc family of adapter proteins is well known for its role in growth factor signaling, especially the Ras/MAPK pathway, but a role in RANKL expression and osteoclast formation has not been shown previously. Hermanns et al. have shown that mutation of OSMR Tyr^861^ in transfected COS-7 cells not only decreased Shc1 binding to the OSMR, but also decreased phosphorylation of ERK stimulated by OSM without affecting STAT phosphorylation (26). These data suggest that Shc1 plays a unique role in downstream signaling induced by OSM.

In this report, we show for the first time that Shc1 is expressed in osteoblasts and that OSM, but not LIF, upregulates the mRNA expression of *Shc1*. Furthermore, OSM, but not LIF, induced the phosphorylation of Shc1. Furthermore, OSM consistently induced phosphorylation of the p52 isoform, which is the isoform known to be important for activation of the Ras/MAPK pathway. By silencing the expression of *Shc1*, it was found that activations of both STAT3 and ERK elicited by OSM were substantially decreased. Activation of Shc1, however, was not affected by silencing of *Stat3*, indicating that STAT3 is acting downstream of Shc1 in osteoblasts. Although some reports have shown that Shc1 is not upstream of STAT3, a recent study in breast cancer cells expressing *Shc1* mutated in the domain containing Tyr^239^ and Tyr^240^ has also demonstrated that Shc1 is upstream of STAT3 (29). The present observations confirmed previous finding showing that activation of STAT3 is part of the downstream signaling which occurs following LIF and OSM receptor activations, but the more robust activation of STAT3 by OSM and the unique activation of ERK by OSM suggest that these particular effects are dependent on recruitment of Shc1 to the OSMR. In addition, by using the siRNA approach to decrease the expression of *Shc1*, it was shown that recruitment of Shc1 to the OSMR was a crucial downstream signaling event in the mechanism by which OSM induces *Tnfsf11* mRNA expression and osteoclast formation. Using the same technique, it was also found that induction of *Tnfsf11* mRNA expression in osteoblasts, and osteoclast formation in co-cultures stimulated by OSM, were critically dependent on the expression of *Stat3* mRNA.

**Figure 8:**
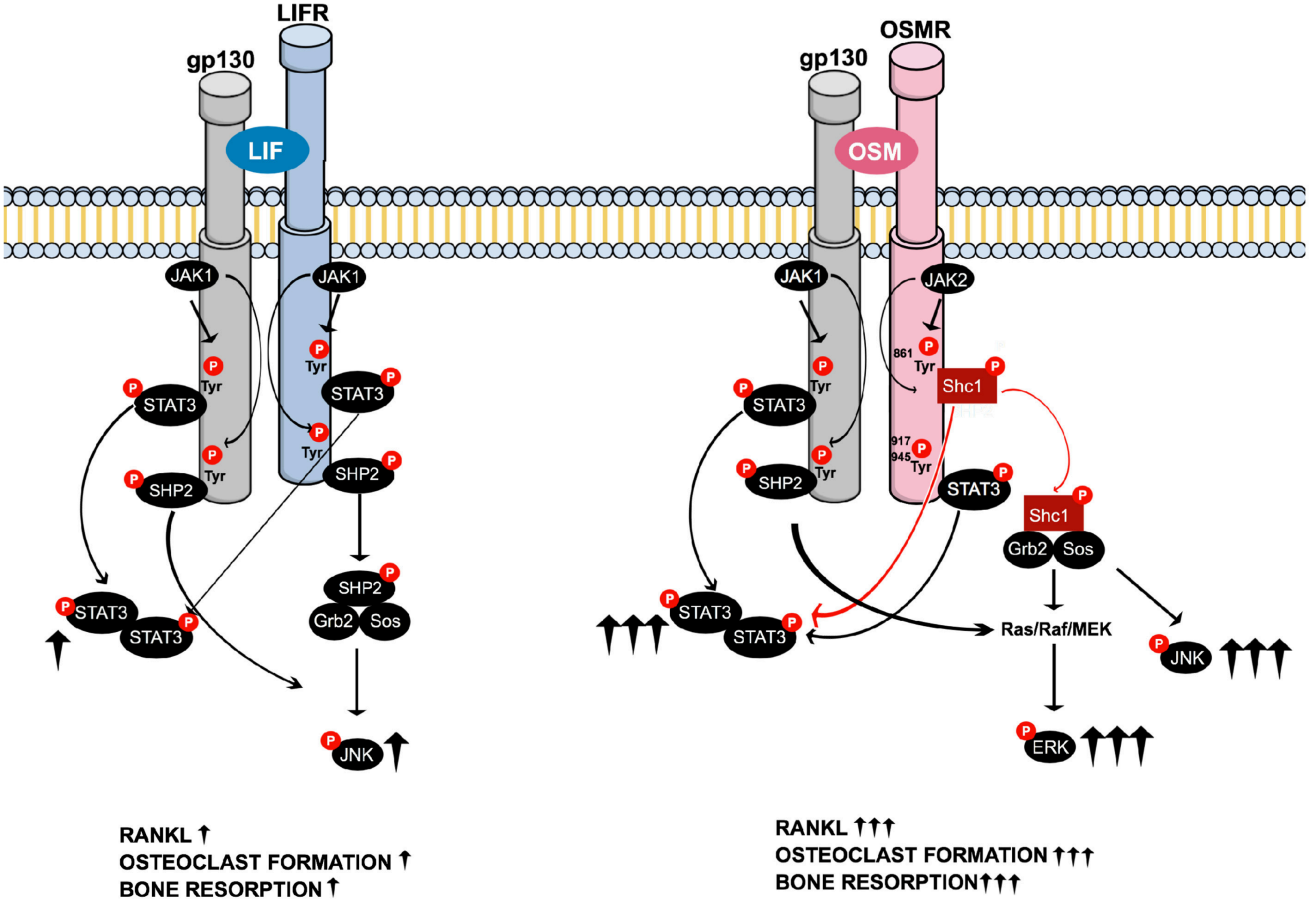
Schematic drawing of the suggested intracellular signaling pathways of LIF and OSM receptors in osteoblasts mediating expression of osteoclastogenic factors, osteoclast formation and bone resorption. Common LIFR/OSMR signaling pathways induced by JAK-mediated phosphorylation of the signal transducer gp130 involve activation of STAT3, as well as activation of JNK through a possible SHP2/Ras/Raf/MAPK cascade **(Left)**. The figure indicates that LIF binds to the LIFR. As discussed in the Introduction section, 2nd paragraph, OSM can also bind to the LIFR to regulate sclerostin expression. In addition to the gp130-mediated pathways common for LIFR and OSMR, the OSM receptor has previously been shown to activate a Shc1-mediated pathway where phosphorylation of the OSMR on Tyr^861^ results in docking and phosphorylation of the adapter molecule Shc1 (26, 27). The activated pShc1 is recruited to the Grb2:SoS complex which in turn induces a Ras/Raf/MAPK cascade that ultimately activates ERK. The Shc1-mediated signaling pathway **(Right)** is suggested to explain the stronger effects by OSM on expression of osteoclastogenic factors, osteoclast formation and bone resorption in comparison to activation of the LIFR:gp130 complex by LIF.

In summary, when compared to LIF, OSM was found to be a more potent stimulator of calvarial bone resorption and osteoclast formation in bone marrow cultures. This was due to greater stimulation of RANKL expression in osteoblasts/stromal cells caused by enhanced activation of STAT3 and JNK by OSM and an ability of OSM to activate ERK that was not shared by LIF. These novel findings in the present study show that the robust and unique stimulatory effects of OSM are dependent on the recruitment of the activated adapter protein Shc1 to the OSMR subunit of the OSMR:gp130 heterodimer, a recruitment that is absent in the LIFR:gp130 complex. From a clinical perspective, inhibition of Shc1 could be a mechanism to decrease OSM-induced bone loss in inflammatory diseases. The suggested differences in signaling downstream of the OSMR and LIFR complexes are summarized in Figure 8.

## Data Availability

The raw data supporting the conclusions of this manuscript will be made available by the authors, without undue reservation, to any qualified researcher.

## Ethics Statement

This study was carried out in accordance with the recommendations of Institutional Animal Care and Ethics Committees at Umeå University, at the School of Dentistry, Araraquara and at the University of Gothenburg guidelines. The protocol was approved by the Institutional Animal Care and Ethics Committees at Umeå University, at the School of Dentistry, Araraquara and at the University of Gothenburg.

## Author Contributions

EP and PS contributed equally. EP, PS, and UL designed the study. EP, PS, TF-M, and PH conducted the experiments. EP, PS, TF-M, PH, HC, and UL interpreted the data. EP, PS, and UL wrote the first draft of the manuscript which was edited and approved by all authors.

### Conflict of Interest Statement

The authors declare that the research was conducted in the absence of any commercial or financial relationships that could be construed as a potential conflict of interest.

## Abbreviations

AMV: avian myeloblastosis virus
BMM: bone marrow macrophages
BMC: bone marrow cells
BMP-2: bone morphogenetic protein-2
D3, 1: 25(OH)_2_-vitamin D3
EMSA: electrophoretic mobility shift assay
GAPDH: glyceraldehyde-3-phosphate dehydrogenase
gp130: glycoprotein 130
Grb2: Growth factor receptor-binding protein 2
IL: interleukin
JAK: Janus kinase
LIF: leukemia inhibitory factor
LIFR: LIF receptor
MAPK: mitogen-activated protein kinase
M-CSF: macrophage colony-stimulating factor
OPG: osteoprotegerin
OSM: oncostatin M
OSMR: OSM receptor
PTB: phosphotyrosine binding domain
PTH: parathyroid hormone
RANKL: receptor activator of NF-κB ligand
SH2: Src homology 2
Shc: Src homology and collagen
SHP-2: SH2 domain-containing tyrosine phosphatase 2
Sos: Son of sevenless
STAT: signal transducer and activator of transcription
TRAP: tartrate-resistant acid phosphatase
TRAP^+^MuOCL: TRAP^+^ multinucleated osteoclasts.
