# Supplementary material for: Activation of Shc1 Allows Oncostatin M to Induce RANKL and Osteoclast Formation More Effectively Than Leukemia Inhibitory Factor
Source: Front Immunol. 2019 May 28;10:1164. doi: 10.3389/fimmu.2019.01164 (PMC6547810; doi:10.3389/fimmu.2019.01164)
Supplement: Supplementary file 1 [file Data_Sheet_1.PDF]

Supporting information Figure S1

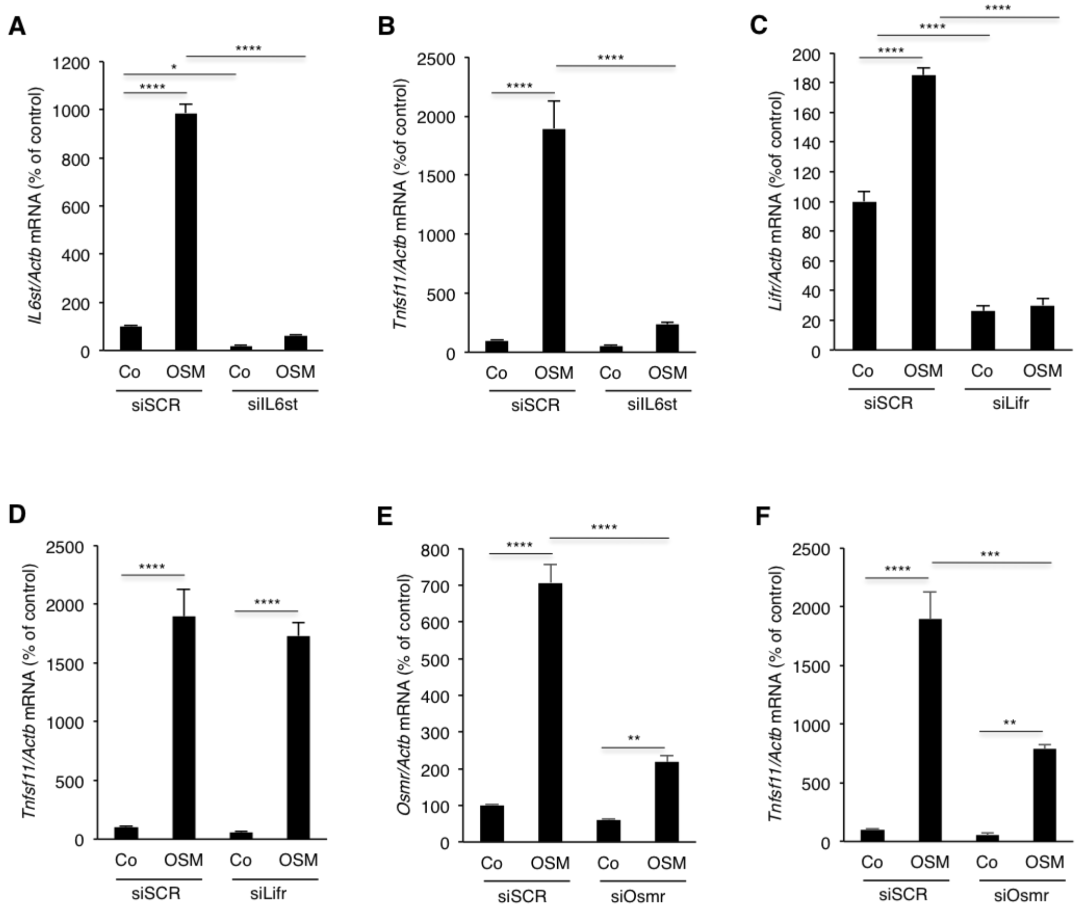

Figure S1. The stimulatory effect of OSM on *Tnfsf11* mRNA is dependent on the expression of *Il6st* and *Osmr*, but independent of *Lifr* expression. Calvarial osteoblasts in which *Il6st* (*A, B*), *Lifr* (C, *D*) or *Osmr* (E, F) were knocked-down by siRNA were treated with OSM at 100ng/mL or vehicle for 24h before analysis of *Tnfsf11* gene expression. Osteoblasts transfected with scrambled RNA (SCR) were treated similarly and analysed. Values represent means for four wells and SEM is shown as vertical bars. Significant differences are indicated by horizontal lines where *, $p < 0.05$; **, $p < 0.01$; *** $p < 0.001$; ****, $p < 0.0001$. (Ordinary two-way ANOVA followed by Tukey post-test).

Supporting information Table I

Antibodies and conditions for the use in western blot experiments.

Specificity (clone no)	Residue phosphorylated	Company	Cat. number	Dilution	Diluent	Amount of protein	Band size
**Mouse IgG**	-	Santa Cruz	sc-2005	1:5000	BSA 1%	-	-
**Rabbit IgG**	-	Santa Cruz	sc-2004	1:5000	BSA 1%	-	-
**Goat IgG**	-	Santa Cruz	sc-2768	1:5000	BSA 1%	-	-
**ERK1 (C-16)**		Santa Cruz	sc-93	1:100	BSA 1%	3μg	44kDa
**p-ERK (E-4)**	Tyr 204	Santa Cruz	sc-7383	1:100	BSA 1%	3μg	42/44kDa
**pSHC (Y427)**	Tyr 427	Abcam	ab68166	1:1000	BSA 1%	4μg	66/52/46kDa
**pSTAT3 (Y705)**	Tyr 705	Abcam	ab76315	1:10000	BSA 1%	3μg	88 kDa
**SHC (H-108)**	-	Santa Cruz	sc-1695	1:100	BSA 1%	4μg	66/52/46 kDa
**STAT3 (9D8)**	-	Abcam	ab119352	1:1000	BSA 1%	3μg	86/91 kDa
**Vinculin (SPM227)**	-	Abcam	Ab18058	1:1000	BSA 1%		124kDa
**β-actin (I-19)**	-	Santa Cruz	sc-1616	1:5000	BSA 1%	3μg	42kDa

Supporting information Table II

RefSeq number and ID assay for the Taqman assays (Applied Biosystems) used to detect the target genes.

Target gene	RefSeq	ID Assay
IL6st	NM_010560.3	s68298
LIFR	NM_001113386.1<br>NM_013584.2	s69223
OSMR	NM_011019.3	s71149
SHC1	NM_001113331.2<br>NM_011368.5	s73682
STAT3	NM_011486.4<br>NM_213659.2<br>NM_213660.2	s74451
Negative Control	-	AM4635

Supporting information Table III

Sequences of primers and probes, annealing temperature (Ta) used for the PCR reaction, numbers of the 5' and 3' ends, GenBank accession numbers and number of base pairs and of the predicted PCR products

Target gene	Sequences	5' and 3' ends	GenBank	Base pair	Ta
36B4 (real-time)		649-710	BC_011106	62	
Sense	5'-CCCTGAAGTGCTCGACATCA-3'				
Antisense	5'-TGCGGACACCCTCCAGAA-3'				
Probe	VIC-AGAGCAGGCCCTGCACTCTCGC-TAMRA				
Acp5 (real-time)		771-847	BC019160	77	
Sense	5‘-CGACCATTGTTAGCCACATACG-3‘				
Antisense	5‘-TCGTCCTGAAGATACTGCAGGTT-3‘				
Probe	VIC-CACTGCCTACCTGTGTGGACATGA-TAMRA				
Acp5 (RT-PCR)		1072-1384	NM_007388	313	58
Sense	5‘-AAATCACTCTTCAAGACCAG-3‘				
Antisense	5‘-TTATTGAACAGCAGTGACAG-3‘				
Actb (real-time)		471-563	M12481	93	
Sense	5'-GGACCTGACGGACTACCTCATG-3'				
Antisense	5'-TCTTTGATGTCACGCACGATTT-3'				
Probe	VIC-CCTGACCGAGCGTGGCTACAGCTTC-TAMRA				
Alpl (RT-PCR)		882-1251	X13409	369	61
Sense	5´-CATCAGTATTTGGAAGAGCTTTAA-3'				
Antisense	5'-AACCACAGTCAAGGTGTCTTTCT-3'				
Fos (RT-PCR)		1667-2348	V00727	277	57
Sense	5'-CCAGACCTGCAGTGGCTGGTGCAGC-3'				
Antisense	5'-CTGTCAGCTCCCTCCTCCGATTCCG-3'				
Jun (RT-PCR)		981-1105	NM_010591	125	57
Sense	5'-AGAGCGGTGCCTACGGCTACAGTAA-3'				
Antisense	5'-CGACGTGAGAAGGTCCGAGTTCTTG-3'				
Ctsk (real-time)		606-695	NM_007802	90	
Sense	5'-ATATGTGGGCCAGGATGAAAGTT-3'				

Antisense	5’-TCGTTCCCCACAGGAATCTCT-3’				
Probe	VIC-CCACGGCAAAGGCAGCTAAATGCA-TAMRA				
Ctr (real-time)		689-764	NM_007588	76	
Sense	5’-AGTTGCCCTCTTATGAAGGAGAAG-3’				
Antisense	5’-GGAGTGTCGTCCCAGCACAT-3’				
Probe	VIC-TCTGTACTGCAACCGCACCTGGGA-TAMRA				
Ctr (RT-PCR)		1483-1650	U185421	167	64
Sense	5’-TGCTGGCTGAGTGCAGAAACC-3’				
Antisense	5’-GGCCTTCACAGCCTTCAGGTAC-3’				
Fos (RT-PCR)		1250-1555	X14897	306	55
Sense	5’-TCATCACCCTCCGCCGAGTCTCAGT-3’				
Antisense	5’-GCTTGCCCCGCCAGTGCTGTAG-3’				
Fosl1 (RT-PCR)		1108-3499	AF017128	294	55
Sense	5’-GACCAGACTCCGAGAGGC-3’				
Antisense	5’-GATAGGCCAGAGGTCGGG-3’				
Fosl2 (RT-PCR)		59-387	X83971	329	57
Sense	5’-CTGCGCACGCCGAGTCCTACT-3’				
Antisense	5’-GATTCGACGCTTCTCCTCCTCCTCA-3’				
Gapdh (RT-PCR)		957-1223	M32599	267	57
Sense	5’-ACTTTGTCAAGCTCATTTCC-3’				
Antisense	5’-TGCAGCGAACTTTATTGATG-3’				
Il6st (RT-PCR)		330-632	X62646	303	57
Sense	5’-TCATCAACAGAACCCGTCC-3’				
Antisense	5’-CCATACATGAAGTGCCATGC-3’				
Junb (RT-PCR)		5536-5944	U20735	409	55
Sense	5’-CCGGATGTGCACGAAAATGGAACAG-3’				
Antisense	5’-ACCGTCCGCAAAGCCCTCCTG-3’				
Jund (RT-PCR)		246-566	NM_010592	321	57

Sense	5'-GAGCAGCATGCTGAAGAAAG-3'				
Antisense	5'-AGCTGGCTTTGCTTGTGCAG-3'				
Lifr (RT-PCR)		2595-2758	NM_013584	164	55
Sense	5'-GCTGTCATTGTTGGCGTGG-3'				
Antisense	5'-TTCATTTCCAATGTCTTAAGAGC-3'				
Osmr (RT-PCR)		2571-2990	NM_011019	420	57
Sense	5'-GATGTACCCACTAAGCCGCC-3'				
Antisense	5'-GAGGACCGTTGAGGTCAAGC-3'				
Shca (real-time)		60-169	NM_011368.5	109	60
Sense	5'-GGTCCTGGGGTGAAAGTT-3'				
Antisense	5'-TGTTCATGTCCAGGGTCTCA-3				
Tnfsf11b (real-time)		845-958	U94331	114	
Sense	5'-AGCTGCTGAAGCTGTGGAA-3'				
Antisense	5'-GGTTCGAGTGGCCGAGAT-3'				
Probe	VIC-CCAAGACATTGACCTCTGTGAAAGCA-TAMRA				
Tnfsf11b (RT-PCR)		428-1147	NM_008764	720	
Sense	5'-TGGAGATCGAATTCTGCTTG-3'				
Antisense	5'-TCAAGTGCTTGAGGGCATAC-3'				
Tnfsf11 (real-time)		606-680	AF053713	75	
Sense	5'-TGGAAGGCTCATGGTTGGAT-3'				
Antisense	5'-CATTGATGGTGAGGTGTGCAA-3'				
Probe	VIC-AGGCTTGCCTCGCTGGGCCAG-TAMRA				
Tnfsf11 (RT-PCR)		929-1739	AF013170	810	
Sense	5'-GGTCGGGCAATTCTGAATT-3'				
Antisense	5'-GGGAATTACAAAGTGCACCAG-3'				

Supporting information Table IV

RefSeq number and ID assay for the Taqman assays (Applied Biosystems) used to detect the target genes.

Target gene	RefSeq	ID Assay
IL6st	NM_010560.3	Mm00439665_m1
Lifr	NM_001113386.1<br>NM_013584.2	Mm00442942_m1
Osmr	NM_011019.3	Mm01307326_m1
Stat3	NM_011486.4<br>NM_213659.2<br>NM_213660.2	Mm01219775_m1
